# Lentinan confers protection against type 1 diabetes by inducing regulatory T cell in spontaneous non-obese diabetic mice

**DOI:** 10.1038/s41387-023-00233-7

**Published:** 2023-04-08

**Authors:** Tijun Wu, Zhi Cai, Fandi Niu, Bin Qian, Peng Sun, Nan Yang, Jing Pang, Hongliang Mei, Xiaoai Chang, Fang Chen, Yunxia Zhu, Yating Li, Fu-Gen Wu, Yaqin Zhang, Ting Lei, Xiao Han

**Affiliations:** 1 Key Laboratory of Human Functional Genomics of Jiangsu Province, Department of Biochemistry and Molecular Biology, Nanjing Medical University, Nanjing, 211166 China; 2 Department of Neurosurgery, Tongji Hospital, Tongji Medical College, Huazhong University of Science and Technology, Wuhan, 430030 China; 3 State Key Laboratory of Natural Medicines, School of Life Science and Technology, China Pharmaceutical University, Nanjing, 2111198 China; 4 Department of Biochemistry and Molecular Cell Biology, Shanghai Jiao Tong University School of Medicine, Shanghai, 200001 China; 5 Department of Pharmacy, Nanjing Drum Tower Hospital, the Affiliated Hospital of Nanjing University Medical School, Nanjing, 210008 China; 6 State Key Laboratory of Bioelectronics, School of Biological Science and Medical Engineering, Southeast University, Nanjing, 210096 China

**Keywords:** Type 1 diabetes, Type 1 diabetes

## Abstract

**Background:**

Lentinan (LNT) is a complex fungal component that possesses effective antitumor and immunostimulating properties. However, there is a paucity of studies regarding the effects and mechanisms of LNT on type 1 diabetes.

**Objective:**

In the current study, we investigated whether an intraperitoneal injection of LNT can diminish the risk of developing type 1 diabetes (T1D) in non-obese diabetic (NOD) mice and further examined possible mechanisms of LNT’s effects. *Methods:* Pre-diabetic female NOD mice 8 weeks of age, NOD mice with 140–160 mg/dL, 200–230 mg/dL or 350–450 mg/dL blood glucose levels were randomly divided into two groups and intraperitoneally injected with 5 mg/kg LNT or PBS every other day. Then, blood sugar levels, pancreas slices, spleen, PnLN and pancreas cells from treatment mice were examined.

**Results:**

Our results demonstrated that low-dosage injections (5 mg/kg) of LNT significantly suppressed immunopathology in mice with autoimmune diabetes but increased the Foxp3+ regulatory T cells (Treg cells) proportion in mice. LNT treatment induced the production of Tregs in the spleen and PnLN cells of NOD mice in vitro. Furthermore, the adoptive transfer of Treg cells extracted from LNT-treated NOD mice confirmed that LNT induced Treg function in vivo and revealed an enhanced suppressive capacity as compared to the Tregs isolated from the control group.

**Conclusion:**

LNT was capable of stimulating the production of Treg cells from naive CD4 + T cells, which implies that LNT exhibits therapeutic values as a tolerogenic adjuvant and may be used to reverse hyperglycaemia in the early and late stages of T1D.

## Introduction

As an autoimmune disease, type 1 diabetes (T1D) can be attributed to the autoreactive destruction of pancreatic β cells, which is caused by T cells and precipitates a decline in insulin production [1, 2]. Among the 230 million patients affected by diabetes worldwide, 4.9 million suffer from T1D; the morbidity of T1D is rising by 3–5% annually on a global scale [3, 4]. Numerous studies have highlighted the use of islet transplantation as a potential mode for T1D treatment [5–7]. However, the widespread utilisation of this procedure is limited by the shortage of adequate islets, reduction of islet cell cluster subsequent to islet extraction, and putative autoimmune destruction of transplanted islets. In addition, the use of immunosuppressive drugs during islet transplantation may lead to side effects [8–10]. Therefore, it is prudent to investigate new and cost-effective drugs for T1D prevention or treatment in order to reduce the morbidity and mortality triggered by this autoimmune disease.

The consumption of mushrooms is an age-old practice in various cultures and is known to be highly beneficial to human health [11]. As one of the most commonly cultivated edible mushrooms, *Lentinus edodes* (shiitake mushroom) exhibits high value for medical applications [12]. Currently, *Lentinus edodes* is used in the treatment of several diseases, such as fungal infections, bronchial inflammation, depressed immune function (like AIDS), frequent flus and colds, cancers, environmental allergies and urinary incontinence [13]. Recent studies have further demonstrated that *Lentinus edodes* has beneficial effects on controlling blood glucose in streptozotocin-stimulated diabetic rats [14, 15]. Lentinan (LNT), the backbone of β-(1, 3)-glucan with β-(1, 6) branches, is an active ingredient extracted from *Lentinus edodes*. More importantly, mounting experimental and epidemiological investigations have reported that LNT can contribute to the decline in the risk of chronic diseases, including gut inflammation [16], chronic hepatitis B infection [17] and cancers [18–21]. However, to our knowledge, no study has investigated whether LNT has any effect on autoimmune-related T1D. To address this issue, the current study set out to determine whether the intraperitoneal administration of LNT can lower the risk for T1D development in non-obese diabetic (NOD) mice, followed by further analysis of the putative mechanism of LNT. We observed that the abdominal administration of LNT depressed immunopathology in models with autoimmune diabetes. LNT was capable of stimulating the production of Treg cells from naive CD4 + T cells, which implies that LNT exhibits therapeutic values as a tolerogenic adjuvant and may be used to reverse hyperglycaemia in the early and late stages of T1D.

## Materials and methods

### Animal models

Female NOD/LtJ mice (aged 5–24 weeks; Jackson Laboratory, Bar Harbor, Maine, United States) were chosen. To detect hyperglycaemia in the obtained mice, glucose levels were monitored using tail vein blood samples from the NOD/LtJ mice that were obtained with Ascensia Microfill blood glucose test strips (Bayer, Mishawaka, Indiana, United States). Animal experimental procedures were implemented under ratification from the Animal and Use Committee of Nanjing Medical University (Permission Number: 20110003), and extensive efforts were conducted to avoid the unnecessary suffering of these mice.

### Treating mice with LNT

Purified LNT was purchased from Jiangsu Yongjian Pharmaceutical Co., Ltd. (Taizhou, China) and diluted with phosphate buffered saline (PBS) prior to injection. All used reagents are listed in Table 1. The 8-week-old female NOD/LtJ mice were randomly divided into 2 groups (*n* = 15, for each group) and intraperitoneally injected with 5 mg/kg LNT in 100 μL PBS or only 100 μL PBS every other day, and subsequent detection of blood sugar levels in mice were carried out. A previous study used LNT at a dosage of 20 mg/kg [22], but we employed an approach of 5 mg/kg LNT injections as a significantly low dose. The mice were euthanized after two consecutive weeks of >200 mg/dL blood sugar levels; all mice were euthanized when >50% control mice developed a disease. To investigate its therapeutic effects on T1D, LNT in PBS was intraperitoneally injected into new-onset diabetic NOD mice [23] (140–160 mg/dL, 200–230 mg/dL or 350–450 mg/dL blood glucose) until the end of the experiment. No blinding was done. The pancreatic lymph nodes (PnLNs), spleens and pancreases were obtained from the mice for histological and fluorescence-activated cell sorting (FACS) experimentation.

**Table 1:** The information of used reagents.

Reagents	Company	Clone
Purified anti-mouse CD3 (no azide and low-endotoxin) antibody	eBioscience	145-2C11
Purified anti-mouse CD28 (no azide and low-endotoxin) antibody	eBioscience	37.51
Fluorochrome-conjugated anti-mouse CD4 antibody	eBioscience	RM4-5
Fluorochrome-conjugated anti-mouse CD8α antibody	eBioscience	53-6.7
Fluorochrome-conjugated anti-mouse CD45 antibody	eBioscience	30-F11
Fluorochrome-conjugated anti-mouse CD25 antibody	eBioscience	PC61.5 and eBio7D4
Fluorochrome-conjugated anti-mouse/rat Foxp3 antibody	eBioscience	FJK-16a
Fluorochrome-conjugated anti-mouse IL-4 antibody	eBioscience	11B11
Fluorochrome-conjugated anti-mouse IL-17A antibody	BioLegend	TC11-18H10.1
Fluorochrome-conjugated anti-mouse IFN-γ antibody	BioLegend	XMG1.2
Fluorochrome-conjugated anti-mouse CD45.1 antibody	Miltenyibiotec	A20
Fluorochrome-conjugated anti-mouse CD45.2 antibody	Miltenyibiotec	104-2

### FACS analysis

Single-cell suspensions were made from spleens, PnLNs and pancreas of NOD mice. Cells were washed and re-suspended in staining buffer (PBS containing 2% bovine serum albumin and 0.05% NaN3). Intranuclear staining was implemented using the method described in the Fixation/Permeabilization buffer solution manuals (eBioscience). Intracellular cytokine staining was implemented by inducing cells at 37 °C with 10 ng/mL phorbol-12-myristate-13-acetate (PMA), 250 ng/mL ionomycin and Golgi-Plug (1:1000; BD Pharmingen) for 4 hours. Next, cell fixing was conducted with a fixation/permeabilization buffer solution (BD Biosciences). The stained cells were acquired with the help of a FACSCalibur flow cytometer (BD Biosciences) before data processing was carried out using FlowJo software (Tree Star, Ashland, Oregon, United States). Specific regions were marked, followed by setting of the gates and quadrants during data analysis according to the isotype control background staining. A minimum of 10,000 cells in each sample were assessed.

### In vitro differentiation of mouse Treg cells

Magnetic cell sorting (Miltenyi Biotec) or FACS sorting (BD FACSAria II) was carried out for the purification of CD4 + CD25- (naive) T cells from mouse PnLNs and spleens. Afterwards, cell incubation was implemented in 24-well plates at 0.4 × 10^6^ cells/well with 1.5 μg/mL plate-bound anti-CD3 and 1.5 μg/mL soluble anti-CD28 at 37 °C. Next, a cell culture was performed with high glucose DMEM encompassing 10% FBS or with the same medium encompassing variable concentrations of LNT (50 μg/mL or 100 μg/mL) subsequent to TCR stimulation. After three days, FACS staining was conducted to analyse the cells.

### CFSE labelling

As previously described, serial dilution of the intracellular dye carboxifluorescein diacetate succinimidyl ester (CFSE) was applied to determine the rates of individual cell proliferation [24]. Specifically, naive CD4 + CD25- T cells were separated from a single suspension of NOD mice splenocytes and PnLNs using microbeads according to the manufacturer’s instructions (Miltenyi Biotech, Auburn, CA). CD4 + CD25- T cells were suspended at 5 × 10^7^/ml in PBS with 5 μM CFSE and incubated at 37 °C for 20 min. Cells were washed with PBS and then incubated in a complete medium encompassing 50 or 100 μg/mL LNT or PBS subsequent to TCR stimulation. Cells were harvested after 24 h, 48 h and 72 h stained for CD4 and analysed by flow cytometry.

### Real-time PCR

Subsequent to total RNA content extraction from cultured cells using RNeasy mini kits (Qiagen), a high-capacity cDNA reverse transcription kit (Applied Biosystems) was used to generate cDNA. Quantitative real-time polymerase chain reaction (PCR) was implemented based on protocols of the TaqMan gene expression assay kits (Applied Biosystems). *Hprt* mRNA functioned as a normaliser for the obtained results.

### In vitro Treg cell suppression assays

CD4 + CD25- (naive) T cells were extracted from spleens and PnLNs in CD45.1 congenic C57BL/6 mice. Subsequently, in the presence of LNT or PBS, FACS sorting (BD FACSAria II) was conducted for a three-day extraction of CD4 + CD25 + Foxp3+ Treg cells from cultured CD4 + CD25-Foxp3-T cells using 1.5 μg/mL plate-bound anti-CD3 and 1.5 μg/mL soluble anti-CD28 (all were CD45.2 + ). Different ratios of CD45.2+ Treg cells were then incubated with CFSE-tagged CD45.1 + CD4 + CD25- T cells in the presence of anti-CD3/CD28 (0.5 μg/mL). Finally, FACS was utilised to analyse CFSE-diluted CD45.1+ effector T cells after a three-day culture.

### Treg-cell transfer experiment

Subsequent to 15-day treatment, total purified Treg cells were obtained from the spleens of both control and LNT-induced mice (1 × 10^5^ cells/mouse), followed by intravenous injection into euglycemic female NOD mice and monitoring as previously mentioned. The pancreases of the mice were attained subsequent to the termination of the experiments for histopathological analyses.

### Histochemical analysis and the examination of inflammatory responses

Subsequent to 10% formaldehyde fixing, the livers, kidneys, hearts and pancreases were made into paraffin-embedded sections (5 µm), followed by haematoxylin-eosin staining. For the pancreas, an analysis of the stained sections was conducted using a grading system in a blinded manner, wherein 0 represented no infiltration, one indicated peri-islet infiltration (<5%), two suggested 5–25% islet infiltration, three represented 25–50% islet infiltration, and four stood for >50% islet infiltration [25, 26]. Approximately 20 islets in each group were assessed. At least three mice were analysed, followed by the calculation of the insulitis score.

### Statistical analysis

The GraphPad Prism 8 software was adopted to process the data. Unless stated otherwise, two different groups were compared with the unpaired two-tailed Student’s *t* test; data among multiple groups were compared using a one-way analysis of variance. T1D progression was analysed using Kaplan-Meier survival curves and log-rank analyses between the cohorts. The total number of infiltrated islets was compared between the test and control groups using a Fisher’s exact test. *p < 0.05 was regarded as statistically significant.

## Results

### LNT suppresses T1D in NOD mice

**Fig. 1:**
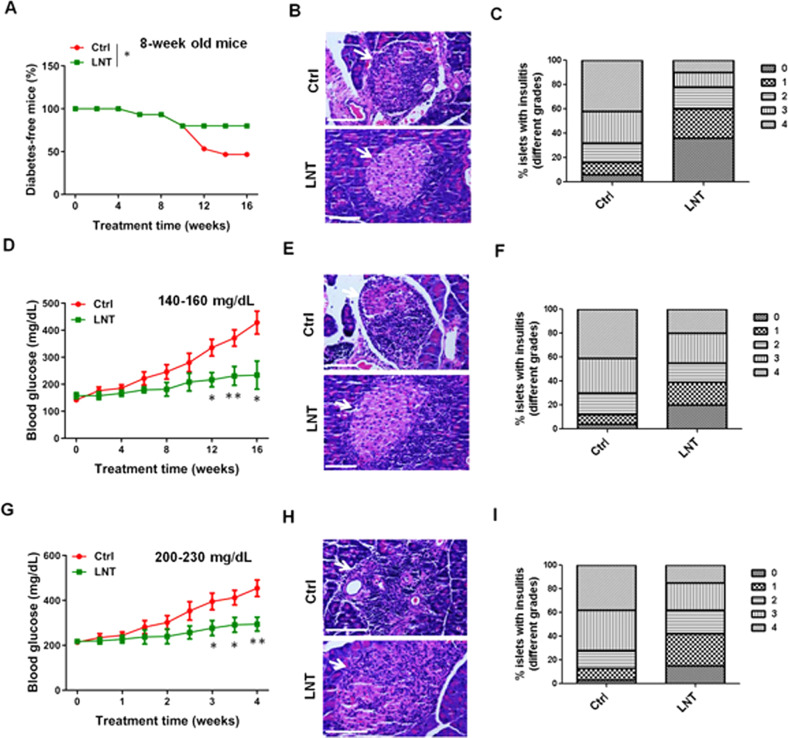
LNT treatment suppresses spontaneous diabetic frequency in NOD mice. Pre-diabetic female NOD mice 8 weeks of age received intraperitoneal treatment with 5 mg/kg LNT in 100 μL PBS (LNT) or only 100 μL PBS (Ctrl) as a control every other day for 16 weeks, followed by an evaluation of T1D development. Hyperglycaemia in mice was measured weekly, and two consecutive weeks of a glucose level >250 mg/dL was considered indicative of diabetes. **A** The frequency of mice without T1D over time (*n* = 15). **B** Representative history pancreas slices from mice in (**A**). Pancreatic islets are indicated by white arrows. **C** The percentages of islets with varying grades of insulitis (*n* = 20). The stages (0–4) represent diabetes progression (also suits for **F** and **I**). NOD mice with 140–160 mg/dL blood glucose levels underwent intraperitoneal treatment with 5 mg/kg LNT before assessment of diabetes progression. **D** Blood glucose levels in mice over time (*n* = 5). **E** Representative histology pancreas slices from the mice in (**D**). Pancreatic islets are indicated by white arrows. **F** The frequency of islets with grade 0–4 insulitis (*n* = 20). NOD mice with 200–230 mg/dL blood glucose received an intraperitoneal treatment with 5 mg/kg LNT before regular monitoring of the diabetes progression. **G** Blood glucose levels in mice over time (*n* = 5). **H** Representative histology pancreas slices from the mice in (**G**). Pancreatic islets are indicated by white arrows. **I** The frequency of islets with grade 0–4 insulitis (*n* = 20). Summary data are presented as the mean ± SEM. *p < 0.05, **p < 0.01 vs the Ctrl group.

To evaluate LNT’s effects on autoimmune diabetes, female NOD mice underwent intraperitoneal injection with a low dose of LNT (5 mg/kg) [22] starting at 8 weeks of age when the mice were still regarded as prediabetic; the inflammatory process was just beginning, but the blood glucose remained within the normal range [27] (Fig. S1A). As depicted in Fig. 1A–C, control NOD mice began to develop diabetes at nearly 16–18 weeks of age (8–10 weeks post-treatment with PBS), and 50–60% were afflicted with diabetes at 24 weeks of age (16 weeks post-treatment with PBS) (Fig. 1A). However, a vast majority of the LNT-treated NOD mice had no diabetes at 24 weeks of age (Fig. 1A).

As evidence of further protection against diabetes, LNT-treated mice presented with a remarkable decline of insulitis and an increase in preserved islets relative to the controls (Fig. 1B, C). Moreover, when NOD mice exhibited 140–160 mg/dL prediabetic blood glucose levels (Fig. S1B) or 200–230 mg/dL new-onset diabetic levels (Fig. S1C) [28], LNT treatment brought about significant amelioration of hyperglycaemia in most mice for approximately 12 weeks (Fig. 1D) or 3 weeks (Fig. 1G) post-treatment initiation. Consistently, LNT-treated mice effectively prevented immune cell infiltration and pancreatic islet destruction (Fig. 1E, F, H, I). LNT treatment once the mice had reached 350–450 mg/dL diabetic blood glucose levels (Fig. S1D) also brought about significant amelioration of hyperglycaemia in most mice for ~2 weeks (Fig. S1E) post-treatment initiation. Additionally, non-diabetic NOD mice that were 24 weeks of age were treated either with or without LNT for 5 months. The results demonstrated that LNT-treated NOD mice did not exhibit any notable changes in body weight (Fig. S1F), behaviour, gross injuries or other signs of treatment toxicity. At the same time, an extensive histopathological examination of the liver, kidney, heart and pancreas sections of LNT-treated NOD mice did not reveal evidence of acute or chronic injury compared with the control mice (Fig. S1G). Taken together, these findings indicate that LNT causes repression of the immunopathology of NOD mice with autoimmune diabetes.

### LNT depresses autoreactive T cells but elevates Treg in NOD mice

**Fig. 2:**
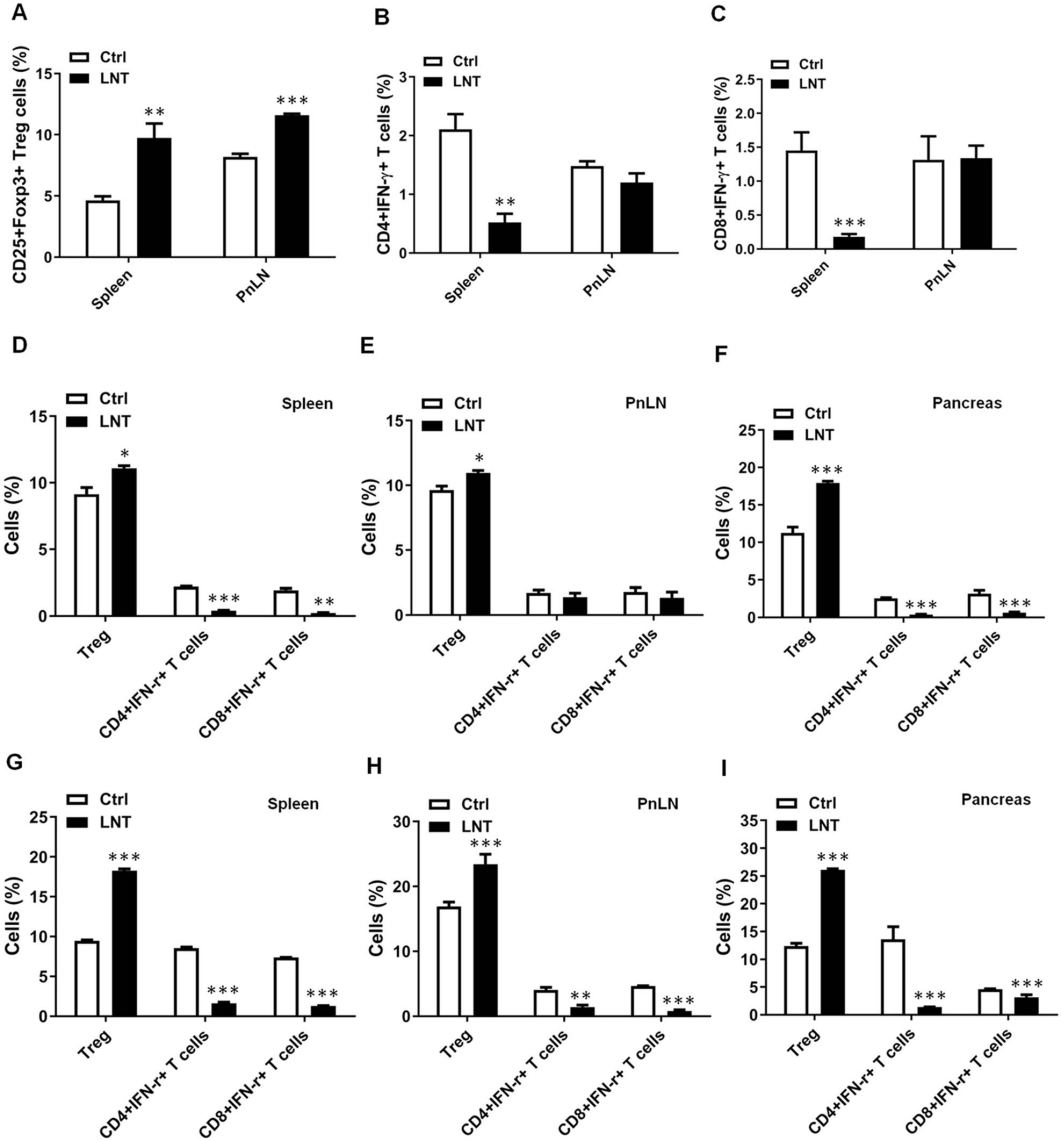
LNT diminishes autoreactive T cells but elevates Treg in NOD mice. Female NOD mice received an intraperitoneal treatment with 5 mg/kg LNT every other day beginning at 8 weeks of age, followed by euthanasia at 24 weeks of age. Manifest CD25 + Foxp3+ Treg cells (**A**), CD4 + IFN-γ + T cells (**B**) and CD8 + IFN-γ + T cells (**C**) frequencies in mice spleens and PnLNs. Foxp3+ Tregs, CD4 + IFN-γ + T cells and CD8 + IFN-γ + T cells frequencies in spleens (**D**), PnLNs (**E**) and pancreases (**F**) of NOD mice with 140–160 mg/dL blood glucose levels treated with LNT or Ctrl for 16 weeks, and blood glucose levels in the Ctrl mice reached 400–500 mg/dL. The frequency of Foxp3+ Tregs, CD4 + IFN-γ + T cells and CD8 + IFN-γ + T cells in spleens (**G**), PnLNs (**H**) and pancreases (**I**) of NOD mice with 200–230 mg/dL blood glucose levels that were treated with LNT or Ctrl for 4 weeks; the blood glucose levels in Ctrl mice reached 450–500 mg/dL. Summary data are shown as the mean ± SEM. *p < 0.05, **p < 0.01, ***p < 0.001 vs the Ctrl group.

NOD mice possess a plethora of immune defects that may result in their autoimmunity expression [27]. Among them, the defective regulatory CD4 + CD25 + Foxp3 + T cells (Tregs) population assumes a pivotal role in T1D induction [29]. To study whether increased Treg cell levels contribute to LNT-orchestrated inhibition of diabetes, spleen, PnLN and pancreas cells from LNT-treated mice (Fig. 1, prediabetic NOD mice aged 8 weeks, NOD mice with 140–160 mg/dL prediabetic blood glucose levels and NOD mice with 200–230 mg/dL new-onset diabetic levels) were examined for Treg cells by means of FACS. As shown in Fig. 2, an obviously enhanced proportion of CD4 + CD25^+^Foxp3^+^ Treg cells existed in the spleens (Fig. 2A, D, G) and PnLNs (Fig. 2A, E, H) of mice treated with LNT compared to the PBS-treated controls. Conversely, LNT treatment reduced CD4 + IFN-γ + (Th1) and CD8 + IFN-γ + T cell frequencies in spleens (Fig. 2B, C, D, G) and PnLNs (Fig. 2H) of the NOD mice. Meanwhile, no difference was found regarding CD4 + IL-17A + (Th17) and CD4 + IL-4 + (Th2) cell frequencies in spleens (Fig. S2A, S2C, S2E) and PnLNs (Fig. S2B, S2D, S2F) between the LNT-treated mice and controls. The pancreases of LNT-treated mice exhibited augmented CD4 + Foxp3+ Treg cell frequency and diminished IFN-γ-producing CD4 + and CD8 + T cell frequencies in contrast to controls (Fig. 2F, I). Collectively, these data indicated that LNT treatment led to potent enhancement in Treg cell frequency and reduction in Th1 cells and CD8 + IFN-γ + T cells in vivo, conferring protection to NOD mice against T1D.

### LNT induces Treg cells

**Fig. 3:**
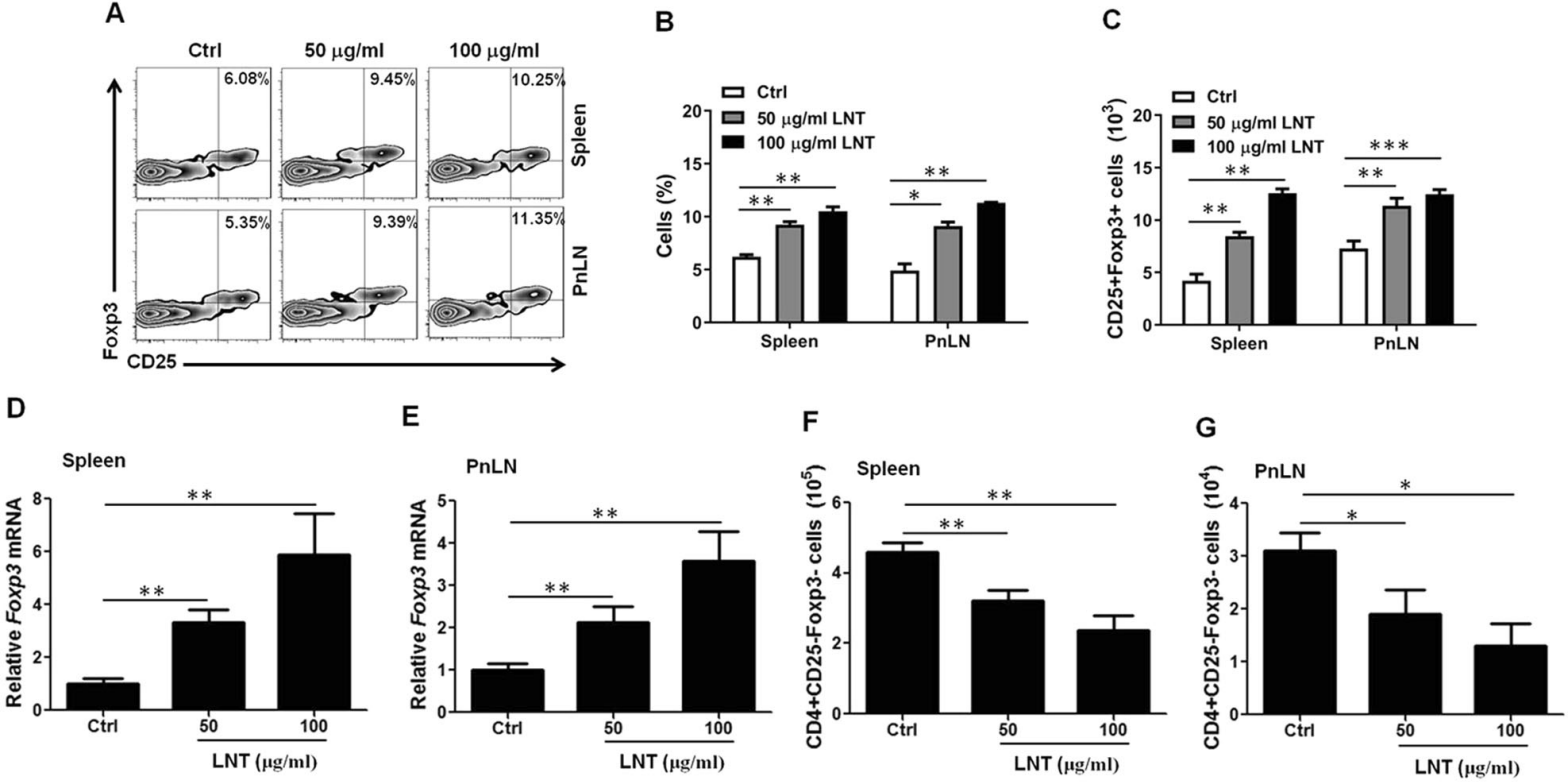
LNT promotes Treg cell differentiation in vitro. CD4 + CD25- (naive) T cells of the spleens and PnLNs from C57BL/6 mice were incubated with anti-CD3 and anti-CD28 for three days in glucose-free DMEM (Ctrl) encompassing 10% FBS-contained or with 50 μg/mL or 100 μg/mL LNT. Representative FACS images (**A**) and CD25 + Foxp3+ Treg cell frequency in CD4 + T cells (**B**) after a three-day incubation. **C** The absolute numbers of CD25 + Foxp3+ Treg cells. The proportion of cells in the gate was suggested by numbers adjacent to outlines in the FACS images. **D**, **E** Foxp3 mRNA levels at 24 h. **F**, **G** Absolute numbers of CD4 + CD25-Foxp3- (Non-Treg) cells. All panels report data verified in at least two independent experiments. Summary data are summarised as the mean ± SEM. *p < 0.05, **p < 0.01, ***p < 0.001.

In order to assess the influence of LNT on T cell activation, naive mouse spleen and PnLN naive CD4 + CD25- T cells were incubated in a medium encompassing 50 or 100 μg/mL LNT or PBS subsequent to TCR stimulation. It was found that LNT influenced neither T-cell activation-related factors nor apoptosis (data not shown). Conversely, LNT diminished T cell proliferation (Fig. S3A), which contributed to a increase in un-proliferated cell numbers subsequent to 48–72 h of incubation. Additionally, LNT-stimulated T cells had decreased levels of mRNAs correlating to type 1 (Th1 cells; Il2 and Ifng) and type 2 (Th2 cells; Il4 and Il13) helper T cells and Il6 compared to control cells, while Treg cells (Il10 and TGF-β1) were augmented with unchanged amounts of Il17a mRNA (Fig. S3B–S3I). Furthermore, LNT generated strikingly enhanced Foxp3+ Treg cells (Fig. 3A–C) and Foxp3 mRNA (Fig. 3D, E) in naive CD4 + CD25- T cells relative to the control group but reduced the absolute number of CD4 + CD25-Foxp3- non-Treg cells at 72 h post-culture (Fig. 3F, G). Together, these findings indicated that LNT specifically accelerated Foxp3 levels and Treg cell fates in naive T cells.

### LNT-induced Treg cells prevent T1D in NOD mice

**Fig. 4:**
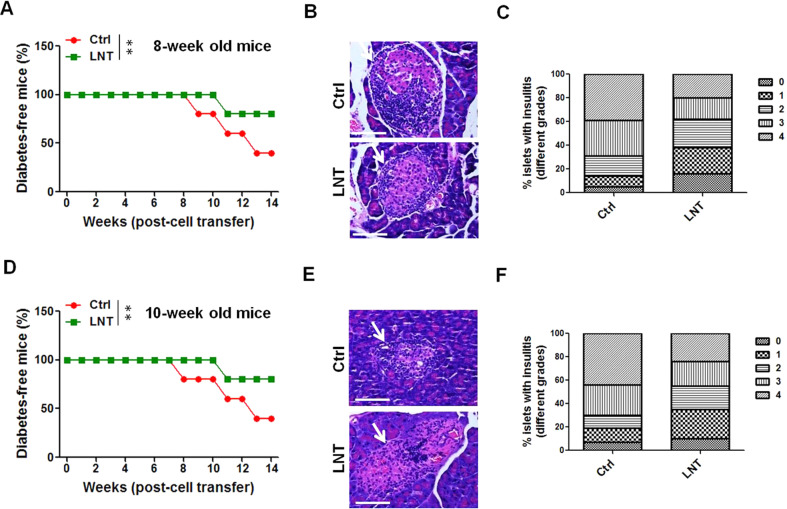
Treatment using LNT-induced Treg cells suppresses the diabetogenicity of NOD mice. Subsequent to the purification of Treg cells from euglycemic PBS (Ctrl) or LNT-treated mice (as depicted in Fig. 1A and in splenic T cells isolated 15 days after receiving the final LNT injection), Treg cells were adoptively transferred to female NOD/Ltj mice at 8 weeks (**A**–**C**) or 10 weeks (**D**–**F**) of age (intravenous; 1 × 10^5^ Treg cells/mouse), followed by weekly monitoring of hyperglycaemia. Mice with two consecutive weeks of 250 mg/dL blood glucose levels were considered to suffer from diabetes. **A** and **D**) The frequency of diabetes-free mice over time. **B** and **E** Representative histology pancreas slices from the mice in (**A**) and (**D**). Pancreatic islets are indicated by white arrows. **C** and **F** The frequency of islets with grade 0–4 insulitis from the mice in (**B**) and (**E**) (*n* = 20). Summary data are summarised as the mean ± SEM. **p < 0.01.

We further analysed Treg cell function following LNT induction and found that in vitro, CD45.1+ effector T cells (Teff) proliferation were appreciably inhibited in LNT-treated CD45.2+ Treg cells (Fig. S4B) compared with PBS treatment controls. In addition, a Treg-cell-transfer model of T1D was applied to ascertain the immunosuppressive role of LNT-treated Treg cells in vivo. Splenic Treg cells extracted from mice euthanized following a 2-week LNT treatment were adoptively transferred into NOD mice aged 8 or 10 weeks (Fig. S4A), followed by an examination of their capability of manipulating ongoing autoimmunity and repressing hyperglycaemia. As depicted in Fig. 4A, protection against T1D was higher in prediabetic mice aged 8 weeks that received Treg cells from the spleens of LNT-induced mice than it was in mice receiving Treg cells from control mice. In addition, mice aged 10 weeks (Fig. 4D) that received Treg cells of LNT-induced mice also developed hyperglycaemia much more slowly than the mice that received the Treg cells from control mice. Further, prominently reduced severe immune cell infiltration and insulitis were detected in the pancreatic islets of mice receiving Treg cells of LNT-inducing mice than in mice receiving Treg cells of PBS-treated controls (Fig. 4B, E). Approximately 60% of islets in mice receiving Treg cells of LNT-induced mice exhibited insulitis grade ≤2, and at least 80% islets of mice receiving Treg cells from PBS showed an insulitis severity grade ≥2 (Fig. 4C, F). In conjunction with the aforementioned observations, these results indicated that LNT triggers a depression of the immunopathology in NOD mice with autoimmune diabetes and also that increased amounts of Treg cells participate in this process.

## Discussion

In the current work, we investigated the role of LNT in T1D development, and our findings revealed that a low-dose administration of LNT (5 mg/kg) noticeably repressed T1D onset in NOD mice. Accordingly, LNT administration effectively attenuated pancreatic insulitis in NOD mice. We also found that LNT treatment markedly augmented CD4^+^CD25^+^Foxp3^+^ regulatory T cells in the spleen, PnLN and pancreas of diabetic NOD mice. It was also determined that LNT led to the generation of Treg cells from naive CD4 + T cells. Taken together, our findings indicate that LNT may be a natural agent that can be putatively adopted to protect against T1D by inducing regulatory T cells in spontaneous NOD mice.

Tremendous amounts of effort made by our peers in the scientific community have availed the structure of β-glucans from different sources (oat, barley and shiitake), highlighting the direct bioactive properties of these polysaccharides [30–32]. It is also known that LNT represents a mushroom β-glucan that exhibits effective antitumor and immunostimulating properties [13, 33]. However, as we know, no works have attempted to determine the potential influence of LNT on preventing or treating T1D. Currently, NOD mouse models are regarded as the gold-standard for studying T1D, which, unlike multiple other models for autoimmunity, provide the advantage of developimg spontaneous disease in a manner that is similar to the same process in humans [34]. Importantly, usage of these models has contributed to various improvements, like the identification of numerous autoantigens and biomarkers that are shared by humans, which has helped to develop many therapeutic targets [35]. Thus, it would be plausible to suggest that the use of NOD mouse models in the present study may offer valuable information suitable for further clinical trials in humans.

A study performed by Karumuthil-Melethil demonstrated that fungal β-glucans exert an immunity-modulating effect on NOD mice [26]. In addition, hyperglycaemia in NOD mice has been shown to be completely delayed following induction with zymosan, another fungal β-glucan, at low (25 μg/mouse) or high (100 μg/mouse) doses [25]. In the current study, we determined that low-dosage intraperitoneal injections of LNT (5 mg/kg) could modulate diabetes development in hyperglycaemic NOD mice. In addition, our findings revealed that these intraperitoneal injections of LNT could further disrupt diabetes progress in NOD mice with new-onset diabetes. Long-term injection with LNT brought about no substantial side effects for NOD mice—a result that could be positively implicated in developing similar clinical therapeutic regimens for T1D in humans.

CD4^+^CD25^+^Foxp3^+^ regulatory T cells, which are generated by the normal thymus as a functionally mature T-cell subpopulation, assume an essential role in self-tolerance maintenance [36, 37]. More importantly, the progression of T1D in NOD mice is orchestrated by the balance between diabetogenic T cells (such as Th1 cells [38], Th17 cells [39], CD8^+^ T cells [40] and regulatory T cells) [41, 42]. Autoreactive T cells, primarily IFN-γ-producing CD4 (Th1) and CD8 (Tc1) T cells, all undergo progressive expansion and cause insulin-producing β-cell destruction in the process of T1D. In an inflammatory microenvironment, the remaining β-cells cannot satisfy the metabolic requirement of insulin generation, ultimately precipitating diabetes.

Previous studies have reported that regulatory T cells are capable of actively repressing diabetes in NOD mice [43]. In contrast, Tregs in the peripheral blood are currently not regarded as a central marker for analysing immune manipulation in T1D [41, 44]. In lieu of expanding on our current understanding, we evaluated whether LNT enhanced CD4^+^CD25^+^Foxp3^+^ regulatory T cells and concluded that LNT triggered an elevation in Treg cell frequencies but a diminishment in IFN-γ-producing T effector cells in the spleen, PnLNs and pancreas of NOD mice compared to untreated controls. Our findings indicated that LNT was capable of inducing Treg cells from naive CD4 + T cells. Moreover, in vitro experiments in this study validated the same idea, wherein naive CD4 + T cells were induced to media containing LNT, which brought about significant generation of Foxp3+ Treg cells in a dose-dependent manner (0–100 μg/mL). Of note, in these culture environments, LNT also attenuated levels of various effector T cell cytokines, like *Il13, Il4*, *Ifng* and *Il6*, whilst it did not give rise to potent changes in *Il17a* mRNA expression.

Although the therapeutic influence of LNT in NOD mice, we were unable to determine the extent to which Treg cells mediate LNT’s impacts on diabetes in our work due to critical functions of this pathway in disease progression and in the limitations of NOD model. Additionally, it is highly possible that LNT may function via other mechanisms to depress diabetes in vivo. A number of cell-surface receptors that initiate immune responses have been identified, such as the toll-like receptor, Dectin-1, scavenger receptors, complement receptor 3 and lactosylceramide. The potential involvement of these receptors in LNT-mediated Treg cell responses remains to be explored in greater detail in our future endeavours.

Altogether, the findings obtained in the current study indicate that LNT, the β-glucan extracted from *L. edodes*, can suppress T1D development in NOD mice, which is likely caused by the preservation of functional β cell mass. Furthermore, LNT could effectively up-regulate CD4^+^CD25^+^Foxp3^+^ regulatory T cells in the spleen, PnLNs and pancreas of NOD mice. Overall, our study provides the basic framework for future clinical trials for a further analysis of the anti-diabetic potential of LNT in T1D humans.

## Supplementary information

Fig. S1, Fig. S2, Fig. S3, Fig. S4

## Data Availability

All data generated and analysed during this study are included in this published article.
