# Supplementary material for: Lentinan confers protection against type 1 diabetes by inducing regulatory T cell in spontaneous non-obese diabetic mice
Source: Nutr Diabetes. 2023 Apr 8;13:4. doi: 10.1038/s41387-023-00233-7 (PMC10082833; doi:10.1038/s41387-023-00233-7)
Supplement: Supplementary file 1 — Fig. S1, Fig. S2, Fig. S3, Fig. S4 [file 41387_2023_233_MOESM1_ESM.docx]

**Supplymentary information**


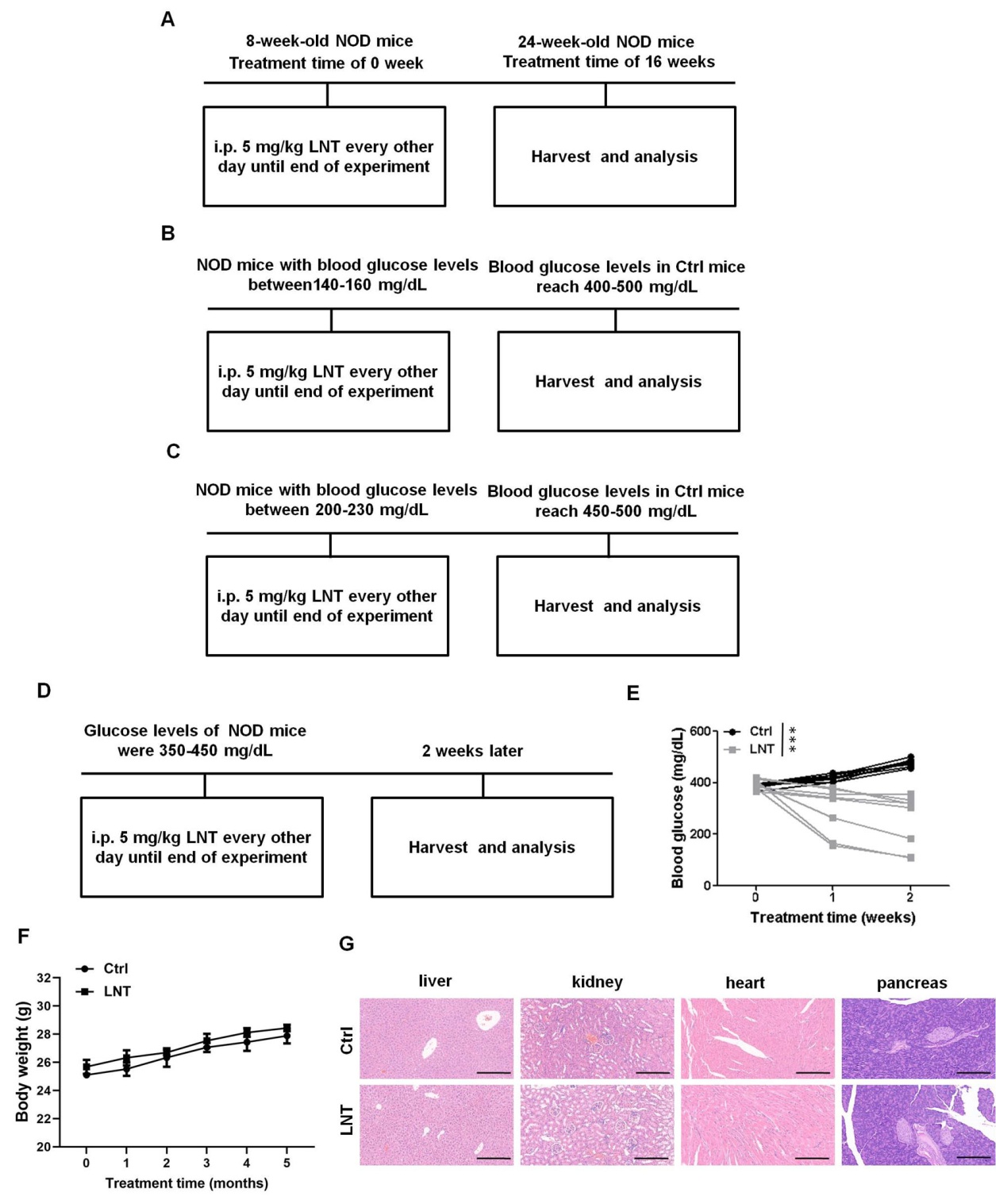


**Fig. S1** LNT ameliorates T1D in NOD mice with new-onset diabetes. **A**) Experimental scheme in a T1D model. **B** and **C**) Experimental scheme in new-onset diabetic NOD mice with 140–160 mg/dL (**B**) and 200–230 mg/dL (**C**) blood glucose levels. **D**) Experimental scheme in NOD mice with 350–450 mg/dL blood glucose. **E**) The blood glucose level of the mice (n = 8). **F** and **G**) Non-diabetic NOD mice underwent intraperitoneal treatment at 24 weeks of age with 5 mg/kg LNT in 100 μL PBS or only 100 μL PBS as a control every other day for 5 months. **F**) The mean body weight was monitored every month and continued up to 44 weeks of age (n = 5). **G**) Representative H&E stained images of paraffin-embedded liver, kidney, heart and pancreas specimens in various treatment mice (×200 magnification). Summary data are presented as the mean ± SEM. *** *p* < 0.001 *vs* the Ctrl group.


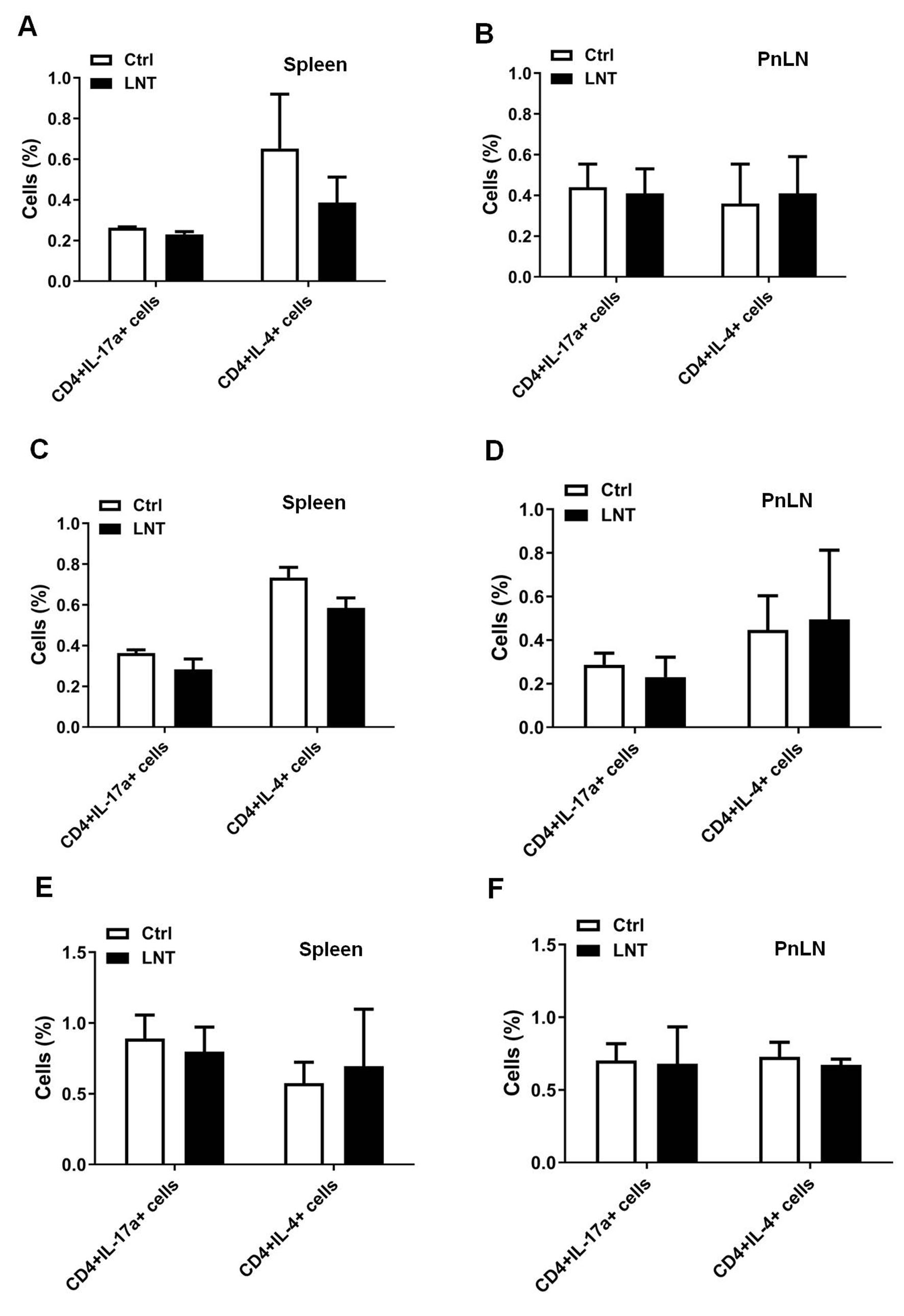


**Fig. S2** LNT does not affect the frequencies of CD4+IL-17a+ T cells and CD4+IL-4+ T cells of spleens and PnLNs from treatment NOD mice. **A** and **B**) Frequencies of CD4+IL-17a+ T cells and CD4+IL-4+ T cells in spleens (**A**) and PnLNs (**B**) of 5 mg/kg LNT-treated NOD mice every other day from 8 weeks of age until euthanasia at 24 weeks of age. **C** and **D**) CD4+IL-17a+ T cell and CD4+IL-4+ T cell frequencies in spleens (**C**) and PnLNs (**D**) of NOD mice with 140–160 mg/dL blood glucose treated with LNT or Ctrl for 16 weeks; the blood glucose levels in Ctrl mice reached 400–500 mg/dL. **E** and **F**) CD4+IL-17a+ T cell and CD4+IL-4+ T cell frequencies in spleens (**E**) and PnLNs (**F**) of NOD mice with 200–230 mg/dL blood glucose levels that were treated with LNT or Ctrl for 4 weeks; the blood glucose levels in Ctrl mice reached 450–500 mg/dL. Summary data are summarized as the mean ± SEM.


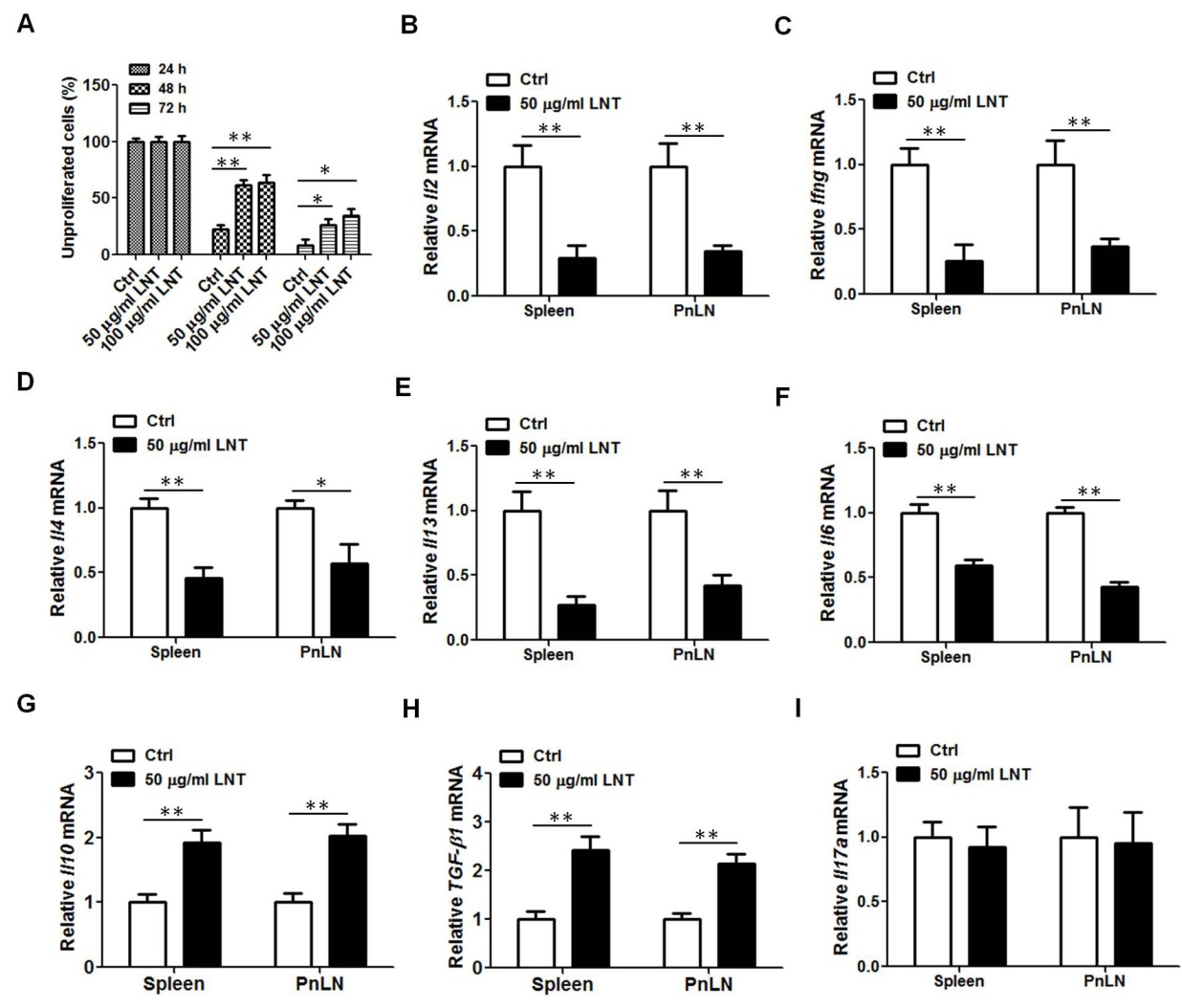


**Fig. S3** LNT suppresses effector T cells. Incubation of CD4+CD25- (naive) T cells from C57BL/6 mice was implemented with anti-CD3 and anti-CD28 for three days using Ctrl or LNT (50 μg/mL or 100 μg/mL) media. **A**) Proliferation by labelling with CFSE dye. The ratio of un-proliferated cells among CD4+ T cells determined after 24, 48 or 72 h. **B**-**I**) Gene expression of il2 (**B**), ifng (**C**), il4 (**D**), il13 (**E**), il16 (**F**), il10 (**G**), TGF-β1 (**H**) and il17a (**I**) at 24 h. All panels report data verified in at least two independent experiments. Summary data are summarized as the mean ± SEM. * *p* < 0.05, ** *p* < 0.01.


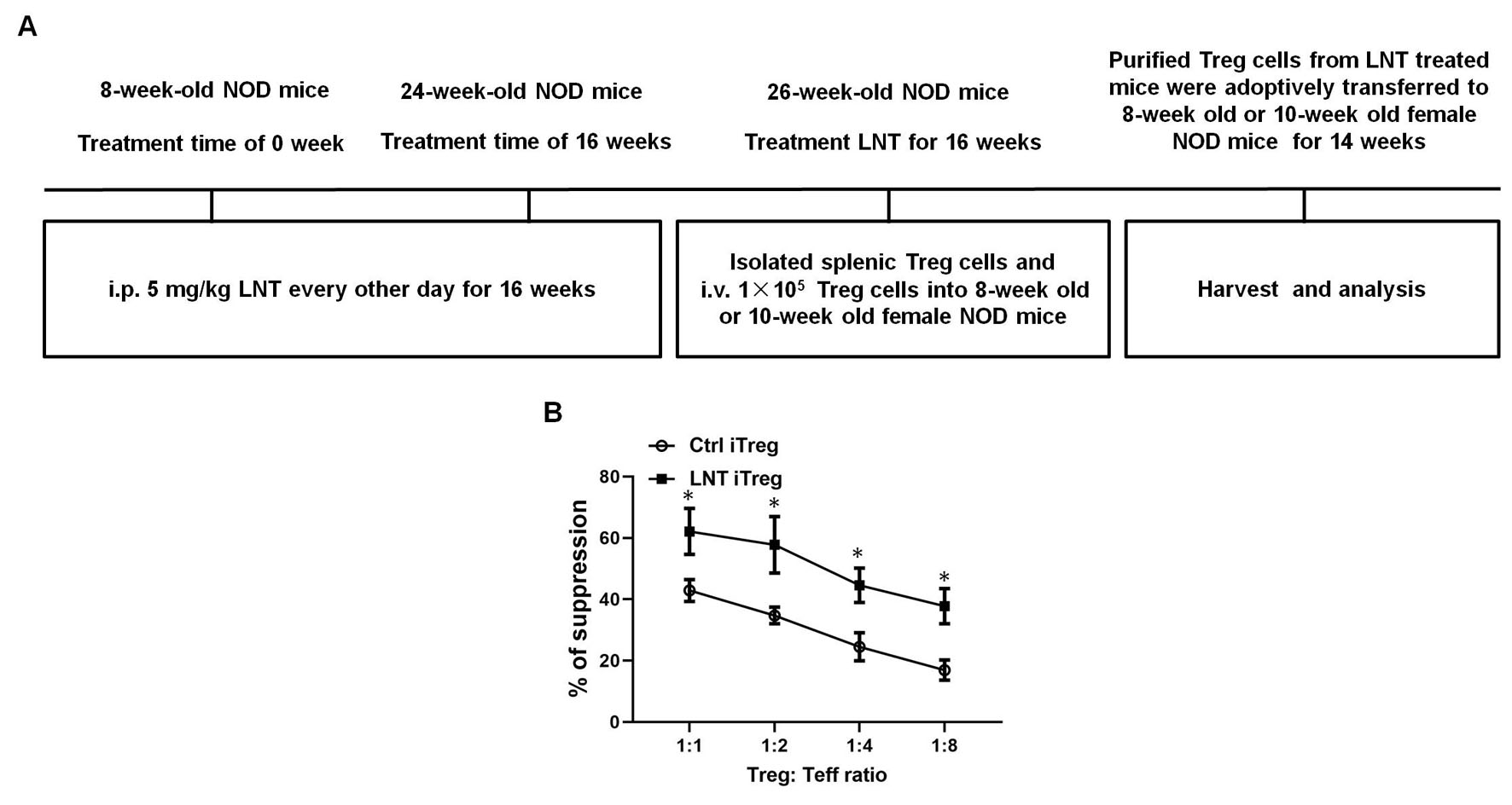


**Fig. S4** LNT-induced Treg cells have suppressive capacity. **A**) An experimental scheme for an adoptive transfer of Treg cells isolated from LNT-induced mice. **B**) LNT-induced CD45.2+CD4+CD25+ Treg (LNT iTreg) or PBS-treated CD45.2+CD4+CD25+ Treg (Ctrl iTreg) from 8-week-old mice were co-cultured with CFSE labelled effector CD45.1+CD4+CD25- T cells (Teff) at the indicated ratios, and the amount of un-proliferated Teff in culture was determined after three days. All panels report data verified in at least two independent experiments. Summary data are summarized as the mean ± SEM. * *p* < 0.05.
